# LED Photobiomodulation for Pain Reduction in Temporomandibular Disorder: A Randomized, Controlled, and Blinded Clinical Trial

**DOI:** 10.1002/jbio.70243

**Published:** 2026-03-06

**Authors:** Lucía Píriz Trindade, Maria Jose Urruty, Laura Hermida Bruno, Federico Todeschini, Luis Eduardo Pascuali Moya, Valentina Lestido, Tainá Caroline dos Santos Malavazzi, Lara Jansiski Motta, Sandra Kalil Bussadori, Kristianne Porta Santos Fernandes, Anna Carolina Ratto Tempestini Horliana, Raquel Agnelli Mesquita‐Ferrari

**Affiliations:** ^1^ Postgraduate Program in Rehabilitation Sciences Universidade Nove de Julho (UNINOVE) Sao Paulo Brazil; ^2^ Universidad Catolica del Uruguay Montevideo Uruguay; ^3^ Postgraduate Program in Biophotonics‐Medicine UNINOVE São Paulo Brazil; ^4^ Department of Stomatology School of Dentistry, University of São Paulo (FOUSP) São Paulo Brazil

**Keywords:** cervical mobility, LED, mandibular mobility, pain, photobiomodulation, temporomandibular dysfunction

## Abstract

This clinical, randomized, controlled, and blinded trial evaluated photobiomodulation (PBM) using 850 nm (infrared) and 660 nm (red) LED clusters on cervical and mandibular mobility and masticatory muscles in temporomandibular disorder (TMD) patients. PBM was applied to the temporomandibular joint (TMJ) region, masseter, temporal, scalene, and trapezius muscles over six non‐consecutive sessions over 2 weeks. The Research Diagnostic Criteria for Temporomandibular Disorders (DC/TMD) were used to determine the presence of TMD. A caliper and goniometer were used to measure the mandibular and cervical range of motion, respectively. Pain was assessed with the visual analog scale (VAS) before and after intervention. Results showed pain reduction only in the LED group post‐intervention; the placebo group showed no difference. No differences were observed in mouth opening, right laterality, flexion, or extension between groups, but left laterality increased in the LED group. In conclusion, PBM with LEDs effectively reduced pain in TMD patients.

**Trial Registration:** ClinicalTrials.gov identifier: NCT06068959

## Graphical abstract

This randomized, controlled, and blinded trial demonstrated that photobiomodulation (PBM) with 850 nm (infrared) and 660 nm (red) LED clusters effectively reduced pain in patients with temporomandibular dysfunction (TMD).

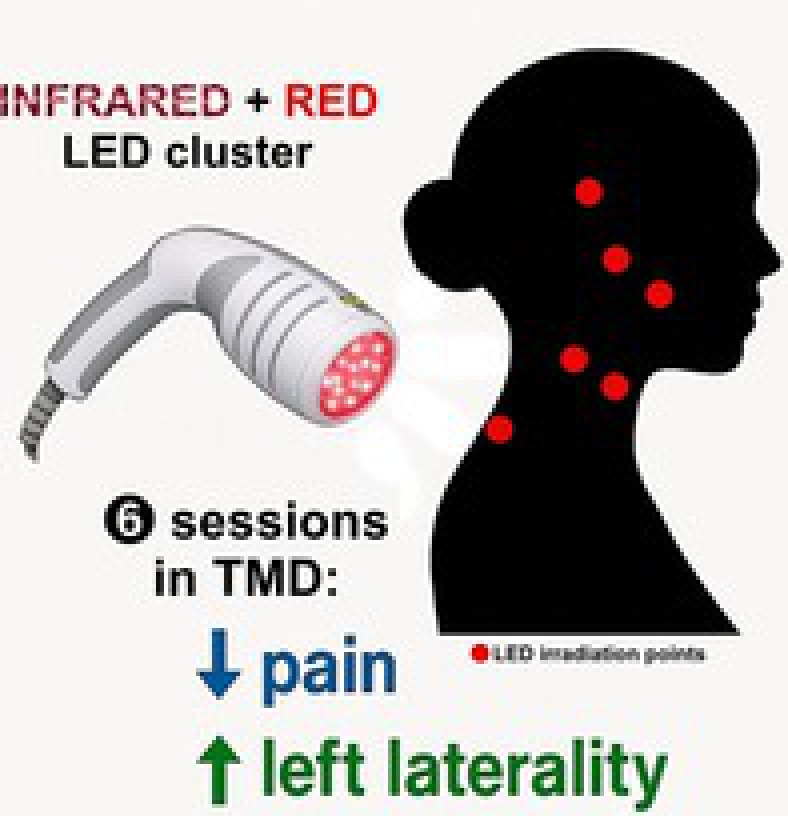

## 1 Introduction

The American Academy of Orofacial Pain defines Temporomandibular disorder (TMD) as a set of disorders that involve the masticatory muscles, the temporomandibular joints (TMJ), and the structures associated with the stomatognathic system. It is the most common cause of orofacial pain, not exclusively of dental origin [1, 2]. TMD occurs in all age groups but is more prevalent in adults, with a higher incidence in women [3].

TMD are characterized by three main signs and symptoms: muscle or joint pain, joint sounds, and/or restriction and deviation of the jaw opening pattern [2, 4]. Among the instruments for the evaluation of TMD, there are questionnaires, clinical assessments, and imaging tests (radiography, computed tomography, and magnetic resonance), which are used based on their applicability and the means to which the patient can access [5].

The Research Diagnostic Criteria for Temporomandibular Disorders (DC/TMD) is often used as a validation and diagnostic instrument, providing clear and precise parameters for data collection and diagnosis [1]. It presents a specific double‐axis system for the diagnosis of TMD, where information is collected on the physical and psychosocial aspects of those evaluated, serving as an organized structure for TMD research [5]. It is currently used worldwide in research involving TMD in clinical studies, diagnoses related to psychosocial factors, and epidemiological studies [6, 7, 8].

In addition to the signs and symptoms mentioned above [9], it is added that painful manifestations, muscular incoordination, and biomechanical imbalance may occur in the cervical region of those with reported TMJ.

Eriksson et al. [10] suggest that functional mandibular movement's result from the coordinated action of the mandibular and cervical muscles, leading to simultaneous movements in the temporomandibular joint. Considering the occipital junction, the Wiesinger atlas shows a strong relationship between TMD and neck pain.

Due to the complexity of TMD, some authors propose treatment associating different forms of therapy and the involvement of a multidisciplinary team [11], including muscle relaxation plates [12], physiotherapy treatments with the use of electrotherapy [13], massage therapy, mobilization resources [14], and photobiomodulation [15, 16, 17].

Photobiomodulation (PBM), also known as low‐level laser therapy (LLLT), is the application of red and infrared light to stimulate healing, alleviate pain, and reduce inflammation. PBM has demonstrated its physiological role as a biostimulatory process, promoting vasodilation, analgesia, anti‐inflammatory effects, and expediting healing processes. Additionally, LED therapy, particularly in patients with fibromyalgia, complements these benefits [15].

Based on the results of studies found in the literature, related to the reduction of pain in patients with temporomandibular disorders, which used PBM with low‐level laser therapy (LLLT) as a therapeutic resource [18], studies were carried out that evaluated, in addition to the pain, also improvement in the range of mandibular movements in these patients [16, 19], or the LED [8, 20], or in inflammatory processes induced in the TMD and with the use of LED as a resource [21], or even with the use of different light sources sometimes in the same device [22]. At the same time, an improvement has also been demonstrated in pain scales and ranges of cervical mobility once low‐level laser therapy was applied [19, 23]. However, there are many protocols with different results and dosimetric parameters, and a need to establish the best way to use the light to obtain the best results, especially using cluster equipment, which allows treatment of the affected regions with different light sources, including red and infrared lights.

Therefore, the objective of this study was to assess the effects of PBM using a cluster of LEDs on pain reduction and functional outcomes in patients with temporomandibular disorders (TMD). Specifically, it aims to determine if PBM significantly reduces pain levels compared to placebo treatment, while also evaluating its impact on mouth opening, cervical inclination (right and left), flexion, and extension.

## 2 Methods

### 2.1 Description of Trial Design

This randomized, controlled, and blind clinical trial was conducted at the Universidad Católica del Uruguay, in the city of Montevideo. The project has the approval from the local human research ethics committee (process number: 230821). The project has been previously registered on the ClinicalTrials.gov website https://clinicaltrials.gov/ under the number NCT06068959. The Consolidated Standards of Reporting Trials (CONSORT) were followed to ensure study quality and transparency.

### 2.2 Eligibility Criteria for Participants

#### 2.2.1 Sample Description

The study had a total of 29 patients previously selected according to the inclusion criteria based on DC/TMD. The objectives and procedure were explained to all participants and carried out. They completed two informed consents. First, they had permission to perform a diagnosis, and those who show signs and symptoms of dysfunction are invited to participate in the study, having them sign a second consent form.

### 2.3 Inclusion/Exclusion Criteria

Patients between 18 and 45 years, with all permanent teeth, and diagnosed with TMD according to DC/TMD (Axis I and II). It excluded individuals with no diagnosis for TMD (Axis I and II), undergoing orthodontic treatment, or undergoing other treatment for TMD, patients with dental caries or gingival disease, and those who initiated or used any type of medication during the phases of the study and patients with comorbidities.

### 2.4 Validation Instruments

Diagnostic Criteria for Temporomandibular Disorder—DC/TMD (AXIS I AND AXIS II).

This validation instrument comprises a questionnaire encompassing inquiries related to general health, oral health, facial pain history, limitations in mouth opening, headaches, noises, habits, tinnitus, joint problems, and current behavior [24].

Following questionnaire completion, a clinical examination was conducted, evaluating mouth opening patterns, vertical extension of mandibular movement, TMJ sounds upon palpation, extrusive movements, and TMJ noises in protrusion and lateral extrusion.

The clinical diagnosis was categorized into three groups:

Group I—Diagnosis of myalgia and arthralgia, including myalgia, local myalgia, myofascial pain, referred myofascial pain, arthralgia, and headache attributed to TMD.

Group II—Intra‐articular disorders, including disc displacement with reduction, disc displacement with reduction and intermittent lock, without reduction with limited opening, and disc displacement without reduction without limited opening.

Group III—Degenerative joint disease.

Upon completion of both components, a diagnosis is derived, ranging from no diagnosis to a maximum of 5 (a diagnosis from group I + a diagnosis from group II + a diagnosis from group III) for each joint. Additionally, in AXIS II, numerical data and scales will be obtained, capturing various psychosocial aspects [25].

### 2.5 Randomization

Randomization was conducted using the online platform https://www.sealedenvelope.com/ employing a 1:1 block randomization scheme. Opaque envelopes were identified with ordinal sequential numbers. The information of the corresponding experimental group will be inserted inside, also sealed. The generation of the random sequence and the preparation of the envelopes were carried out by a person not directly involved in the study. After inclusion of the patient in the study, the investigator who applied PBM opened one envelope (without changing the numerical sequence) and performed the indicated procedure or its simulation. The author of the research was responsible for the storage (digital platforms) and tabulation of information, being an impartial statistician responsible for analyzing the data obtained at the end of the research. These same envelopes containing the patient files (with an anamnesis sheet and informed consent) were kept in a locked file.

### 2.6 Interventions

#### 2.6.1 Clinical Sequence of the Clinical Trial

Participants underwent two phases of data collection. The initial phase, termed the Control Phase, involves a 1‐week period where participants are validated and do not receive any physiotherapy or dental intervention.

Subsequently, the treatment phase was divided into two groups:

− Group 1—LED, which received red and infrared LEDs
− Group 2—PLACEBO, comprising individuals who received applications from an inactive PBM device.

### 2.7 Implementation of Blinding

The study involved a dentist, a dental intern, and a 1 nurse. The current study was characterized as blinded, with blinding occurring only with the patient. The principal investigators (dentists) performed all procedures, including assessments, PBM treatment, and data analysis, which were not blinded during data collection and analysis, knowing to which groups the patients belonged.

Participants were uninformed about the interventions. Participants experienced a simulated PBM in the control group, ensuring a rigorous blinding approach throughout the study. The Statistician remained blinded to the treated groups; the statistician solely received information on Group 1 and Group 2 through sheets.

### 2.8 Photobiomodulation Using an LED Cluster

**FIGURE 1:**
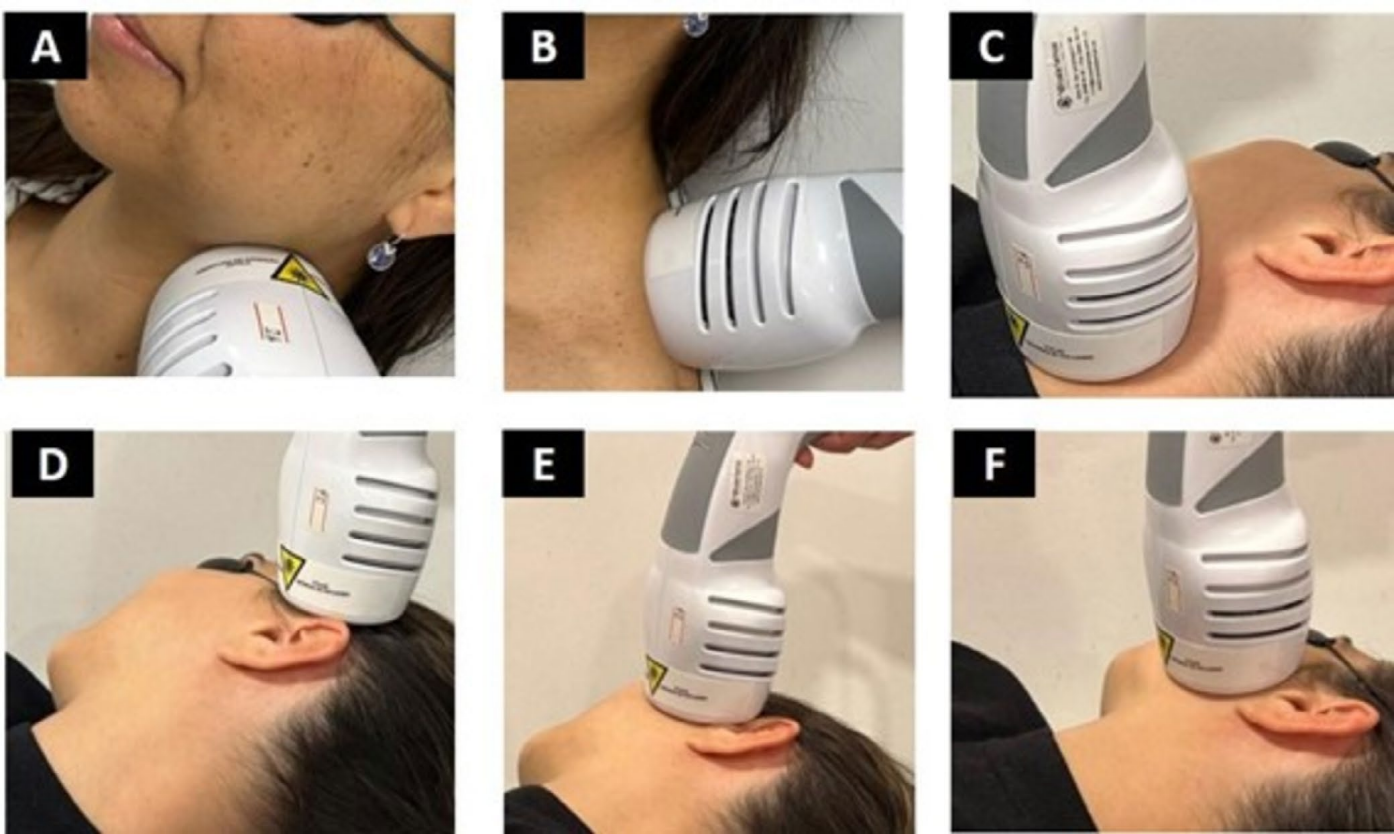
Irradiation regions (bilateral) with PBM using red and infrared LEDs. (A) Scalene muscle, (B) trapezius muscle, (C) sternocleidomastoid muscle, (D) temporal muscle, (E) temporomandibular joint, and (F) masseter muscle.

PBM using red and infrared LEDs was applied bilaterally to the temporomandibular joint regions and the masseter, temporal, sternocleidomastoid, trapezius, and scalene muscles (Figure 1). The treatment consisted of 6 non‐consecutive sessions over 2 weeks, using the Antares LED Cluster (Ibramed, Amparo, SP, and Brazil). The dosimetric parameters were selected based on the study conducted by De Souza et al. [26] and are described in Table 1.

**TABLE 1:** Dosimetric parameters with LED cluster.

Parameters	Values/treatment
Wavelength [nm]	850 infrared 660 red
Operating mode	Continuous
Radiant power [mW]	300 each infrared LED (total cluster 1200) 250 each red LED (total cluster 1250)
Cluster irradiance [mW/cm^2^]	60 infrared 62.5 red
Cluster area [cm^2^]	20
Exposure time [s]	83 infrared 80 red
Radiant exposure [J/cm^2^]	5
Radiant energy [J] per cluster	4 infrared LEDs: 99.6 J (each LED 24.9 J) 5 red LEDs: 100 J (each LED 20 J) Total: 44.9 J per cluster application (infrared + LED) Considering the 6 bilateral regions: 538.8 J per session
Number of irradiated areas with a cluster	Total points in the cluster: 9 points (5 red and 4 infrared). 6 bilateral regions irradiated with the cluster. Points irradiated per treatment: 54 points (108 bilateral points). Regions submitted to cluster irradiation: Temporal Temporomandibular joint Masseter insertion Sternocleidomastoid Scalene Trapezius
Application technique	In contact, at 90° with the surface
Number of sessions and frequency	6 non‐consecutive sessions

For the placebo group, the same procedures were followed as with the LED group, except that the equipment was turned off. The interventions were carried out at the University Health Clinic (UCU). Both the professional and the patient wore protective glasses. The LED cluster was covered with transparent disposable PVC plastic for hygiene and to prevent cross‐contamination. Before local irradiation, a facial cleaning with 70% alcohol was performed. The patient remained in a supine position during the applications.

### 2.9 Outcomes

#### 2.9.1 VAS Visual Analogue Scale

The visual analogue scale (VAS) is a validated instrument allowing us to quantify the current intensity of pain. Consisting of a 10 cm straight line, one end features a verbal description of “no pain,” while the opposite end features another description indicating “intense pain.” Participants were guided to draw a perpendicular line between these two extremes to signify the intensity of the pain they were experiencing. The patient made a mark on a line from 0 to 10 cm, which was then measured in millimeters [26].

#### 2.9.2 Mandibular Range of Motion

A digital caliper was used to validate the range of motion of mandibular movement in millimeters, including openings, left and right lateral movements, and protrusion. This procedure is part of the DC/TMD validation [24].

#### 2.9.3 Cervical Range of Motion

The goniometer was used for the examination of the cervical range of motion, which was performed with the patient sitting to stabilize the pelvis and dorsolumbar area. The patient must maintain body alignment, as any compensation can distort the results obtained. The cervical spine movements included, in the frontal plane, right lateral inclination and left lateral inclination, and in the sagittal plane, flexion and extension [27]. These measurements were performed before commencing the PBM treatment and once it had been concluded.

A demographic pencil was used to mark the anatomical points. Subsequently, with the assistance of the researcher, the patient performed the indicated movements in a passive way. To record flexion‐extension, position 0 was chosen with the goniometer at 90°. The axis was placed over the external auditory canal, the fixed arm aligned with the vertical midline of the head, and the mobile arm at the level of the nostrils.

For both left and right lateral inclination, position 0 with the goniometer was set at 0. The axis was placed on the spinous process of C7, the fixed arm aligned with the vertical midline formed by the dorsal spinous processes, and the mobile arm aligned with the midline of the head, referencing the midpoint of the external occipital protuberance.

#### 2.9.4 Statistical Analysis

After assessing normality with the Shapiro–Wilk test, pain outcomes measured by VAS and the movements of mouth opening, flexion, and extension showed a normal distribution, whereas left and right laterality did not. Therefore, two‐way ANOVA with Bonferroni post hoc was used to evaluate differences between the Placebo and LED groups before and after treatment for parametric variables, while a linear mixed model with Bonferroni post hoc was applied for nonparametric variables. Statistical significance was set at *p* ≤ 0.05, and intergroup differences were evaluated considering the 95% confidence interval when comparing baseline and final means of each group.

## 3 Results

A total of 29 patients were included and divided into two groups: 15 were included in the Placebo (Group 1) and 14 were included in the LED treatment (Group 2). The study variables were measured at two time points: the initial time point (before LED or placebo treatment) and the final time point (after LED or placebo treatment).

Table 2 presents the biological sex distribution among patients, revealing a higher prevalence of females in both the LED treatment group and the placebo group. In the overall study population, females constituted a significantly larger proportion (*n* = 23, 79.3%) compared to men (*n* = 6, 20.7%).

**TABLE 2:** Descriptive characteristics of the patients in terms of gender and age.

Group	Biological sex	Frequency	Age
PLACEBO	Female	11	36.27 ± 8.26
	Male	4	35.25 ± 9.90
	Total	15	36 ± 8.56
LED	Female	12	32.25 ± 7.36
	Male	2	28 ± 9.24
	Total	14	31.64 ± 7.46

**FIGURE 2:**
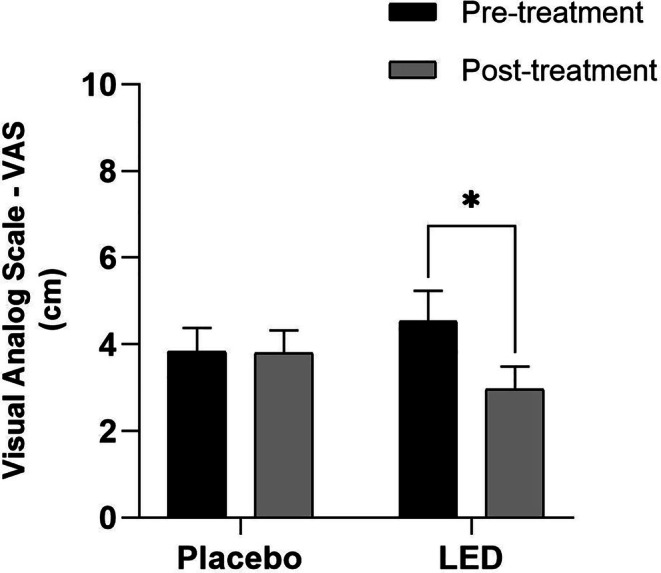
Pain assessment using VAS in the placebo group and active LED group at the beginning and end of the protocol (after 6 weeks). Data expressed in Mean ± SEM (2‐way ANOVA, *p ≤ 0.05).

The results of pain assessment using the VAS scale showed a reduction in pain in the group that received active LED treatment when comparing post‐treatment values with pre‐treatment values (*p* = 0.0009). In contrast, no significant differences in pain were observed in the placebo group (with the LED device turned off) when comparing scores at pre‐ and post‐treatment protocol (Figure 2).

**FIGURE 3:**
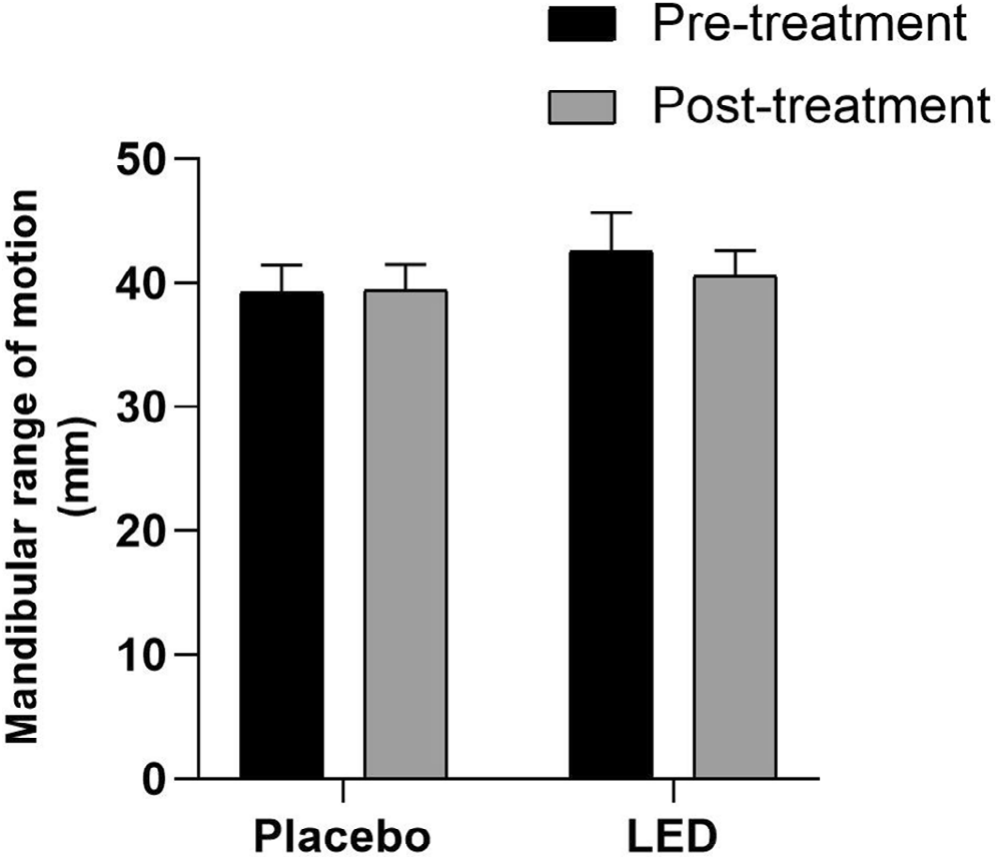
Mean values ± standard error of the mean for the range of motion in mouth opening in the placebo and active LED groups. Data expressed in Mean ± SEM (2‐way ANOVA, Bonferroni, *p ≤ 0.05).

Regarding mandibular opening movements, the results showed no significant differences when comparing the placebo and LED groups before and after the treatment protocol (Figure 3).

**FIGURE 4:**
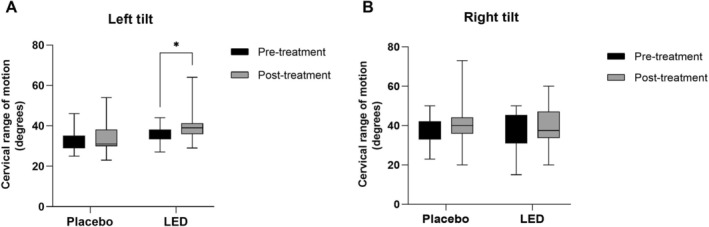
Results expressed as medians, minimum, and maximum values of the cervical range of motion for left tilt (A) and right tilt (B) in the placebo and LED groups at pre‐ and post‐treatment protocol (mixed linear model, Bonferroni, * ≤ 0.05).

The results for the left cervical inclination movement showed an increase in the group that received active LED post‐treatment compared to the values initially evaluated (*p* = 0.0011). In the placebo group, no significant differences were observed when comparing the periods of evaluation (Figure 4A). However, for the right cervical inclination movement, there was no significant difference between the groups when comparing the initial values with those obtained at pre‐ and post‐ of the proposed PBM protocol (Figure 4B).

**FIGURE 5:**
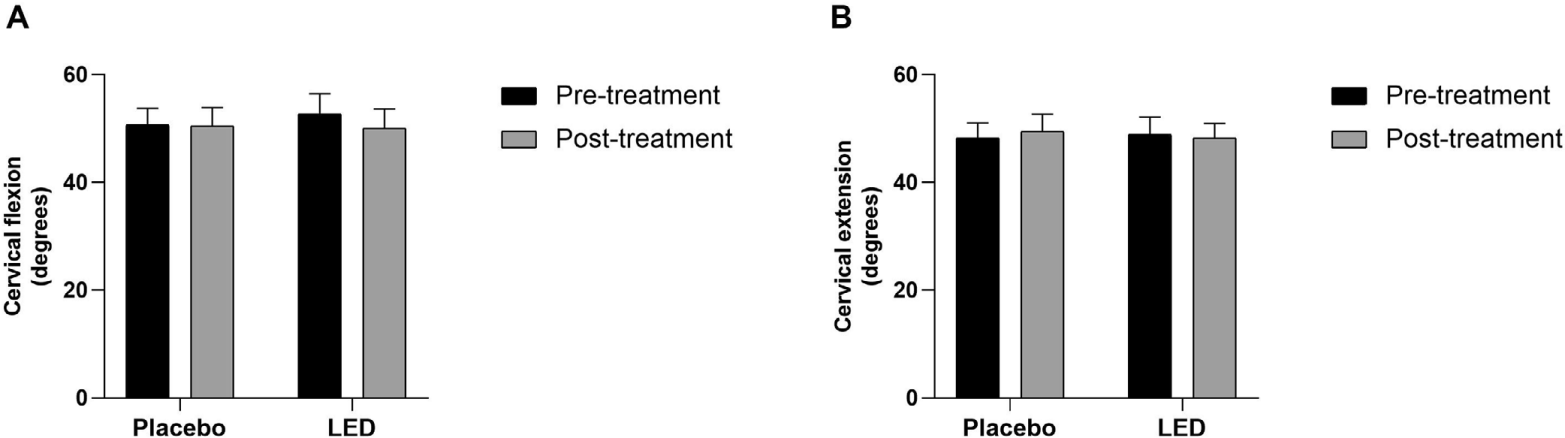
Evaluation of cervical flexion (A) and extension (B) movements in the placebo and LED groups at the pre‐ and post‐treatment protocol. Data expressed in Mean ± SEM (2‐way ANOVA, Bonferroni post hoc, *p ≤ 0.05).

Regarding the cervical flexion and extension, there are no differences between the groups when evaluated pre‐ and post‐treatment (Figure 5).

## 4 Discussion

Temporomandibular disorders (TMD) are the most common cause of non‐dental orofacial pain [1]. TMD occurs across all age groups but is primarily found in adults, with a higher incidence in women [2]. Carrara et al. [4] state that temporomandibular disorders are characterized by three main signs and symptoms: muscle or joint pain, joint sounds, and/or restriction and deviation in the pattern of mandibular opening. In addition to the previously mentioned signs and symptoms [9], they add that painful manifestations, muscle incoordination, and biomechanical imbalance in the cervical region may occur in those with TMD.

Painful processes are very common in patients with TMD, causing considerable discomfort, as well as interfering with their physical and mental function, resulting in lost workdays, reduced productivity, and deterioration in quality of life. The literature has shown that the signs and symptoms of TMD can negatively impact these individuals' quality of life [18, 28, 29, 30]. Consistent with these findings, patients in both groups of the present study reported pain‐related to the presence of TMD at the beginning of the protocol, which was measured using the visual analog scale (VAS).

The literature consistently points to a higher prevalence of TMD in females, with ratios ranging from 3:1 [1], 4:1 [31], and even 6:1 [32], reflecting a clear preference for women in epidemiological data. This trend is corroborated by the observation of a higher incidence in women of childbearing age, at least twice that of men, as seen in previous studies [33, 34]. The role of estrogens has been highlighted as a possible influencing factor in the experience of pain associated with TMD, alongside other biological and psychosocial factors [35]. Additionally, women are twice as likely to report pain‐related symptoms compared to men [36]. Our research also reflects a female predominance, aligning with the results found in the literature. In the total randomized sample we analyzed, we identified a significantly higher prevalence of women (*n* = 23, 79%) compared to men (*n* = 6, 20.68%).

Due to the complexity of TMD, some authors propose treatment that combines different forms of therapy and the involvement of a multidisciplinary team [11], including photobiomodulation (PBM) [16, 17, 18]. From our study, it was possible to verify that treatment with an LED cluster was able to reduce pain assessed by VAS after six sessions, and the difference found when considering pre‐ and post‐treatment was significant compared to the placebo control group, confirming that the effect was indeed caused by the light resources.

The present results align with those of previous studies conducted by De Souza et al. [26] and Panhoca et al. [8], both of which reported significant reductions in pain. In the study conducted by De Souza et al. [26], a device featuring 18 red LEDs (660 nm) and 18 infrared LEDs (850 nm) was utilized, delivering a total irradiated power of 126 mW and 75.6 J per point, administered three times a week over 2 weeks. In contrast, Panhoca et al. [8] employed irradiation parameters of 150 mW, 300 mW/cm^2^, 18 J/cm^2^, and 9 J per point, with treatment provided in eight sessions, twice a week over 4 weeks, resulting in a significant reduction in pain.

The study conducted by Herpich et al. [17] focused exclusively on the immediate effect of PBM using different light sources (LED and LBI) in women. Using 4 LEDs at 640 nm and 4 LEDs at 875 nm, in addition to superpulsed LBI at 905 nm, with a power of 33.4 mW on each side of the face, in a single session with different exposure times (20, 40, and 60 s) irradiating 10 points—five on each side of the face, two points on the masseter and three points on the temporal muscle. Assessments were conducted immediately after, as well as 24 and 48 h later. The results demonstrated a reduction in pain intensity in post‐treatment assessments compared to pre‐treatment in Groups 1, 2, and 3, with a moderate effect size. In our study, assessments were conducted after six sessions, evaluating the effect of treatment after completion and using higher powers (300 and 250 mW for infrared and red LEDs, respectively) and higher energies (44.9 J per application of the cluster). Similarly to the present study, the authors did not find significant effects on mandibular range of motion, aligning with the results of our study.

Al‐Quisi et al. [37] used only red LEDs (660 nm) in patients with TMD, once a week for 4 weeks. They observed a decrease in pain after treatment, but no change in mandibular range of motion, similar to our study. We also observed that clicks in the joint were resolved in all patients (100%) treated with red LEDs, although our study did not aim to evaluate the effect of LEDs on joint clicks. However, the results in terms of pain and range of motion are consistent with those found in the present study.

The literature presents various studies exploring the use of low‐intensity lasers as a light source for pain treatment and improvement of cervical angles in patients with cervical spine disorders. Additionally, studies demonstrated that the use of other types of devices, such as MLS laser therapy (which utilizes two wavelengths simultaneously), in conjunction with mobility exercises, resulted in a decrease in the VAS [38], as well as the use of high‐intensity laser (HILT) combined with transcutaneous electrical nerve stimulation (TENS), which also showed favorable results [39].

However, our study highlights the advantages of using an LED cluster, due to its ease of application, reduced treatment time, and more accessible cost. It is important to note that among the treatment options available for temporomandibular disorders (TMD), light therapy stands out for its non‐invasive nature, absence of discomfort, and low incidence of side effects.

In the present study, we were able to verify through the results of the VAS a significant reduction in pain in patients suffering from pain associated with temporomandibular disorders. These findings suggest that treatment with an LED cluster represents an effective alternative for pain relief. Additionally, we noted an increase in the range of left cervical inclination movements in patients after the application of treatment with the LED cluster. These results reinforce the efficacy and utility of this therapeutic approach to improve the quality of life of patients with TMD.

## 5 Conclusions

In conclusion, the PBM protocol with a cluster of red and infrared LEDs applied to the masticatory and cervical muscles induced a significant reduction in pain after six treatment sessions in individuals with TMD. However, no significant differences were found regarding the range of motion for mouth opening and cervical movements in the group that received the treatment.

## Author Contributions

All authors contributed to the study conception and design. The planning, execution of experiments, and collection were performed by L.P.T., M.J.U., L.H.B., F.T., L.E.P.M., and V.L. The data analysis was performed by L.P.T., T.C.S.M., and R.A.M.‐F. Original draft preparation was written by L.P.T., M.J.U., and T.C.S.M. The review and editing were performed by L.J.M., S.K.B., K.P.S.F., A.C.R.T.H., and R.A.M.‐F. Resources by R.A.M.‐F. Supervision was made by L.J.M., S.K.B., K.P.S.F., A.C.R.T.H., and R.A.M.‐F. Resources by R.A.M.‐F.

## Funding

This work was supported by Conselho Nacional de Desenvolvimento Científico e Tecnológico, 302771/2025‐5 and Fundação de Amparo à Pesquisa do Estado de São Paulo, 2020/13976‐0.

## Ethics Statement

The project received approval from the Research Ethics Committee of Universidad Católica del Uruguay (UCU; Process: 230821).

## Consent

Informed consent was obtained from all individual participants included in the study.

## Conflicts of Interest

The authors declare no conflicts of interest.

## Data Availability

All the data used to support the findings in this study are included in the article.
